# Correlation between thromboembolic risk and prevalence of coronary artery disease in patients with atrial fibrillation and impaired renal function

**DOI:** 10.1016/j.ijcha.2024.101454

**Published:** 2024-07-18

**Authors:** Tsutomu Murakami, Atsuhiko Yagishita, Kengo Ayabe, Susumu Sakama, Kyong Hee Lee, Mari Amino, Koichiro Yoshioka, Yuji Ikari

**Affiliations:** Department of Cardiology, Tokai University School of Medicine, Kanagawa, Japan

**Keywords:** Atrial fibrillation, Coronary artery disease, CHADS_2_ score, Chronic kidney disease

## Abstract

**Background:**

Atrial fibrillation (AF) and coronary artery disease (CAD) often co-occur. The prevalence of coincident AF and CAD, the characteristics of such patients, and the correlation with thromboembolic risk in association with renal function are unclear.

**Methods and Results:**

We studied 435 consecutive patients who underwent contrast-enhanced computed tomography (CT) before catheter ablation for AF. Nineteen patients with inconclusive CT underwent coronary angiography for a calcified coronary artery. Overall, 87 of the 435 patients had CAD (20.0 %: paroxysmal AF, 63.9 %; persistent AF, 35.2 %; and longstanding AF, 0.9 %). Of these, 17.9 % were newly diagnosed with CAD. There was a stepwise increase in CAD prevalence according to the CHADS_2_ score (10.1 % at 0, 20.1 % at 1, 24.7 % at 2, 35.1 % at 3, and 41.7 % at ≥ 4 points). Of note, in patients with low estimated glomerular filtration rate < 50 mL/min/1.73 m^2^, the CAD prevalence increased for all CHADS_2_ scores (15.4 % at 0, 40.0 % at 1, 32.4 % at 2, 38.5 % at 3, and 50.0 % at ≥ 4 points).

**Conclusions:**

The prevalence of coexisting CAD increases with the CHADS_2_ score. This underscores the importance of screening for coexisting CAD in patients who are at high risk for thromboembolic events, particularly in patients with impaired renal function.

## 1 Introduction

Atrial fibrillation (AF) and coronary artery disease (CAD) are both commonly encountered in older patients, and their coexistence is not rare due to shared risk factors [1], [2], [3], [4], [5]. The reported prevalence of CAD in patients with AF ranges from 14 % to 46.5 % [6], [7], [8], [9], [10], whereas the prevalence of AF in patients with CAD ranges from 0.2 % to 5 % [11], [12], [13], [14]. Additionally, patients with new-onset AF are at considerable risk for subsequent new-onset CAD [15]. Coexisting CAD increases the risk of cardiovascular events in patients with AF [16].

The CHADS_2_ score is a stratification scheme used to predict the likelihood of thromboembolic events in patients with AF. The following risk factors are assigned values that guide the administration of anticoagulants: congestive heart failure, hypertension, age ≥ 75 years, diabetes mellitus, and prior stroke or transient ischemic attack [17]. In addition, the presence of chronic kidney disease (CKD) must be confirmed before starting anticoagulant therapy. Considering that patients with high CHADS_2_ scores shared risk factors with those with CAD, we hypothesized that the prevalence of coincident CAD would increase with thromboembolic risk factors in patients with AF. Moreover, considering that patients with CKD are more likely to have CAD [18], the presence of CKD may impact the prevalence of coincident CAD.

Cardiac computed tomography (CT) is commonly used to assess the anatomy of the left atrium and pulmonary veins before catheter ablation for AF. It is a reliable alternative to transesophageal echocardiography, which is considered the gold standard for the detection of left atrial thrombus. Although the prevalence of CAD, detected by cardiac CT before catheter ablation for AF, has been reported to be 41 % and 74 % in two previous studies [19], [20], the relationship between coincident CAD and thromboembolic risk factors has not been fully elucidated. Therefore, this study aimed to investigate the association between CHADS_2_ score and the prevalence of coexisting CAD in association with renal function, assessed using cardiac CT before catheter ablation in patients with AF.

## 2 Methods

### 2.1 Ethical information

This study was approved by the Ethics Committee of Tokai University Hospital (approval no.: 22R-240) and was conducted according to the tenets of the Declaration of Helsinki.

### 2.2 Study population and design

**Fig. 1:**
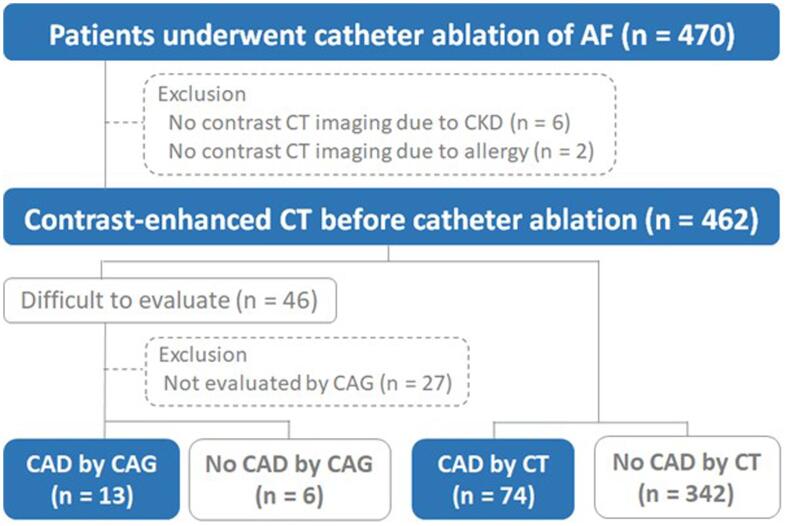
**Patient flowchart.** In total, 470 consecutive patients who underwent catheter ablation were examined retrospectively. Contrast-enhanced CT was performed in 462 patients; it was not performed in eight patients due to a history of hypersensitivity to iodinated contrast media (n = 2) or CKD with a low estimated glomerular filtration rate (n = 6). Another 27 patients were excluded due to lack of consent for CAG; thus, 435 patients were studied overall. CAD was present in 87 of 435 (20.0 %) patients. CT, computed tomography; CAD, coronary artery disease; CKD, chronic kidney disease; CAG, coronary angiography.

We examined retrospectively 470 consecutive patients who underwent catheter ablation for AF. Contrast-enhanced CT was performed in 462 patients; it was not performed in eight patients owing to a previous hypersensitivity reaction to iodinated contrast media (n = 2) or CKD with low estimated glomerular filtration rate (eGFR, n = 6). CAD was defined as stenosis ≥ 50 % on contrast-enhanced CT or coronary angiography and a history of percutaneous coronary intervention (PCI) or coronary artery bypass grafting (CABG), as in a previous study [21]. Coronary angiography was performed in 19 of 46 patients with calcified coronary arteries for whom CT angiography was inconclusive. Informed consent was obtained prior to angiography. After excluding 27 patients who did not undergo diagnostic coronary angiography for calcified coronary arteries, 435 patients were evaluated (Fig. 1).

The following clinical parameters were compared between patients with and without CAD (CAD and no-CAD groups, respectively): age; sex; body mass index (BMI); AF type (paroxysmal, persistent, and longstanding); CHADS_2_ score [17]; smoking history; comorbidities, including sleep apnea, hypertension, diabetes mellitus, and dyslipidemia; medications; laboratory data, including low-density lipoprotein (LDL) cholesterol, high-density lipoprotein (HDL) cholesterol, serum creatinine, eGFR, brain natriuretic peptide (BNP), uric acid, and glycated hemoglobin (HbA1c) levels; left ventricular ejection fraction measured using a modified Simpson’s method; and left atrial diameter measured using M−mode imaging on echocardiography. Persistent AF was defined as episodes lasting > 7 consecutive days, and long-standing persistent AF was defined as episodes lasting > 365 consecutive days [22]. The prevalence of CAD was stratified according to CHADS_2_ scores, and additionally verified in patients with eGFR < 50 mL/min/1.73 m^2^.

### 2.3 Cardiac CT

**Fig. 2:**
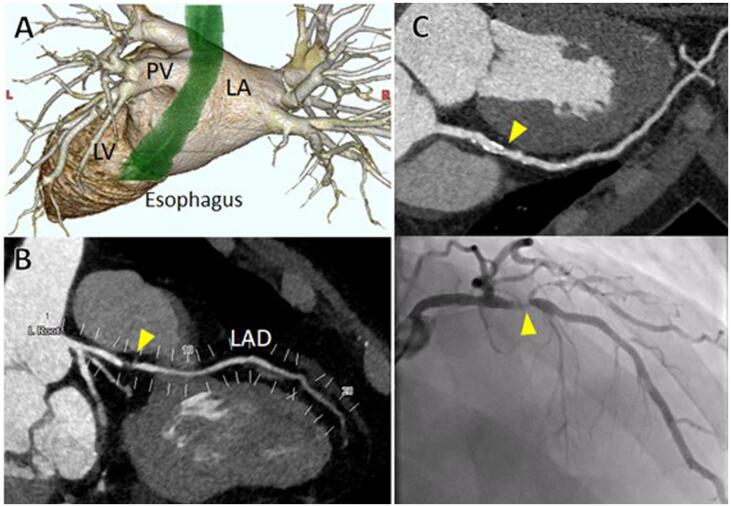
**Representative computed tomography (CT) and coronary angiography images** A. Three-dimensional construction of the left atrium, pulmonary veins, and esophagus (green). LA, left atrium; PV, pulmonary vein; LV, left ventricle. B. A representative case of stenosis in the LAD assessed by axial and cross-sectional images of contrast-enhanced CT in a patient with a CHADS_2_ score of 3 points. Note the severe coronary stenosis in the proximal LAD (yellow arrowhead). LAD, left anterior descending artery. C. A representative case of a calcified LAD that was inconclusive for coronary artery disease on contrast-enhanced CT in a patient with a CHADS_2_ score of 1 point (yellow arrowhead in the upper panel). Coronary angiography reveals stenosis in the LAD (yellow arrowhead in the lower panel). LAD, left anterior descending artery. (For interpretation of the references to colour in this figure legend, the reader is referred to the web version of this article.)

Contrast-enhanced CT images were obtained before catheter ablation with the patient in a supine position using a 192-slice multidetector spiral CT scanner (SOMATOM Force, Siemens, Erlangen, Germany). CT images were obtained during electrocardiographically gated and repeated breath-holds to evaluate the construction of the left atrium and pulmonary veins, the location of the esophagus, and the presence of CAD and to rule out atrial thrombus. The presence of CAD, defined as stenosis ≥ 50 %, was assessed by both expert cardiologists and radiologists and was confirmed by another expert cardiologist when there was disagreement (Fig. 2).

### 2.4 Statistical analyses

Continuous variables with normal distribution are expressed as mean ± standard deviation. Numerical factors with skewed distributions are presented as medians (interquartile ranges). Comparisons of continuous variables with normal distribution were performed using Student’s *t*-test. The Wilcoxon rank-sum test was used to assess differences in the clinical parameters between the two groups. The chi-squared or Fisher’s exact test was used to compare proportions. All statistical calculations were performed using JMP version 14 (SAS Institute Inc., Cary, NC, USA). Statistical significance was set at a p-value < 0.05.

## 3 Results

### 3.1 Baseline characteristics

Table 1 summarizes the baseline characteristics of the 435 patients. Overall, 278 (63.9 %) patients had paroxysmal AF, 153 (35.2 %) had persistent AF, and four (0.9 %) had long-standing AF. Of the 435 patients, 323 were male (74.3 %), and the mean age was 64.7 ± 11.0 years. Mean height, body weight, and BMI were 165.8 ± 9.3 cm, 66.5 ± 13.8 kg, and 24.1 ± 4.1 kg/m^2^, respectively. The mean CHADS_2_ score of all participants was 1.17 ± 1.05; 129 patients (29.7 %) scored 0 points, 164 (37.7 %) 1 point, 93 (21.4 %) 2 points, 37 (8.5 %) 3 points, and 12 (2.8 %) ≥ 4 points.

**Table 1:** Baseline characteristics of studied patients.

	All patients (n = 435)	Patients with CAD (n = 87)	Patients without CAD (n = 348)	P value
Age, years	64.7 ± 11.0	68.5 ± 7.6	63.8 ± 11.5	<0.01
Males, n (%)	323 (74.3)	75 (86.2)	248 (71.3)	<0.01
Height, cm	165.8 ± 9.3	166.4 ± 7.4	165.7 ± 9.7	0.42
Body weight, kg	66.5 ± 13.8	67.4 ± 10.4	66.3 ± 14.5	0.44
Body mass index	24.1 ± 4.1	24.3 ± 3.5	24.0 ± 4.2	0.47
Atrial fibrillation type				0.07
Paroxysmal, n (%)	278 (63.9)	48 (55.2)	230 (66.1)	
Persistent, n (%)	153 (35.2)	37 (42.5)	116 (33.3)	
Long-standing, n (%)	4 (0.9)	2 (2.3)	2 (0.6)	
Heart failure, n (%)	59 (13.6)	19 (21.8)	40 (11.5)	0.01
Hypertension, n (%)	234 (53.8)	64 (73.6)	170 (48.9)	<0.01
Age ≥ 75 years old, n (%)	68 (15.6)	18 (20.7)	50 (14.4)	0.15
Diabetes mellitus, n (%)	76 (17.5)	22 (25.3)	54 (15.5)	0.03
History of stroke, n (%)	37 (10.7)	10 (11.5)	27 (7.8)	0.26
Smoking, n (%)	233 (53.6)	56 (64.4 %)	177 (50.9 %)	0.03
AHI, (available data; n = 309)	13 (6–22)	15 (10–25)	11 (6–20)	0.05
The CHADS_2_ score	1.17 ± 1.05	1.60 ± 1.13	1.07 ± 1.00	<0.01
0	129 (29.7)	13 (15.3)	116 (33.1)	
1	164 (37.7)	33 (36.5)	131 (38.0)	
2	93 (21.4)	23 (26.4)	70 (20.0)	
3	37 (8.5)	13 (15.3)	24 (6.9)	
≥4	12 (2.8)	5 (5.9)	7 (2.0)	
LDL, mg/dL	110 ± 29	99 ± 27	112 ± 29	<0.01
HDL, mg/dL	60 ± 15	56 ± 13	61 ± 16	0.02
Serum creatinine, mg/dL (range)	0.92 (0.78–1.09)	1.05 (0.90–1.25)	0.90 (0.76–1.05)	0.28
Estimated GFR, mL/min/1.73 m^2^	60 ± 16	53 ± 18	62 ± 15	<0.01
BNP, pg/mL (range)	67 (33–139)	80 (46–162)	64 (31–133)	0.03
Uric acid, mg/dL	5.9 ± 1.5	6.2 ± 1.4	5.8 ± 1.5	0.04
HbA1c, %	5.9 ± 0.7	6.1 ± 0.8	5.9 ± 0.7	<0.01
Statin, n (%)	120 (27.6 %)	40 (46.0)	80 (23.0)	<0.01
Diuretics, n (%)	55 (12.6 %)	17 (19.5)	38 (10.9)	0.03
Digoxin, n (%)	13 (3.0)	1 (1.1)	12 (3.4)	0.26
Beta blocker, n (%)	292 (67.1)	67 (77.0)	225 (64.7)	0.03
ACE inhibitor, n (%)	38 (8.7)	11 (12.6)	27 (7.8)	0.15
ARB, n (%)	138 (31.7)	34 (39.1)	104 (29.9)	0.10
Anticoagulant,				
Vitamin K antagonist, n (%)	26 (6.0)	12 (13.8)	14 (4.0)	<0.01
NOAC, n (%)	409 (94.0)	75 (86.2)	334 (96.0)	<0.01
Ejection fraction, %	64.0 ± 11.9	62.4 ± 12.3	64.4 ± 11.8	0.16
Left atrial diameter, mm	40.7 ± 7.6	43.4 ± 7.2	40.0 ± 7.6	<0.01

CAD, coronary artery disease; AHI, Apnea hypopnea index; LDL, low density lipoprotein; HDL, high density lipoprotein; GFR, glomerular filtration; BNP, brain natriuretic peptide; ACE, angiotensin-converting enzyme; ARB, Angiotensin II receptor blocker; NOAC, Non-vitamin K oral anticoagulant.

### 3.2 Prevalence and characteristics of patients with coexisting coronary artery disease (CAD)

Overall, CAD was present in 87 of 435 patients (20.0 %, Fig. 1), including 9 with a history of PCI (n = 8) or PCI and CABG (n = 1). Contrast CT or coronary angiography revealed newly detected CAD in 78 of the 435 patients (17.9 %, Table 2).

**Table 2:** Coronary artery disease.

Coronary artery disease	N = 87
Stenosis of coronary artery (50–75 %)	67
Stenosis of coronary artery (>75 %)	11
History of PCI for stable angina pectoris	7
History of PCI for myocardial infarction	1
History of PCI and CABG	1

PCI, percutaneous coronary intervention; CABG, coronary bypass grafting.

Compared with patients without CAD, those with CAD had higher prevalence of heart failure (21.8 % vs. 11.5 %, p = 0.01), hypertension (73.6 % vs. 48.9 %, p < 0.01), and diabetes mellitus (25.3 % vs. 15.5 %, p = 0.03); higher CHADS_2_ scores (1.60 ± 1.13 vs. 1.07 ± 1.00, p < 0.01); lower eGFR (53 ± 18 mL/min/1.73 m^2^ vs. 62 ± 15 mL/min/1.73 m^2^, p = 0.03); higher serum BNP (80 [46–162] pg/mL vs. 64 [31–133] pg/mL, p = 0.03), serum uric acid (6.2 ± 1.4 mg/dL vs. 5.8 ± 1.5 mg/dL, p = 0.04), and HbA1c (6.1 ± 0.8 % vs. 5.9 ± 0.7 %, p < 0.01) levels; and lower serum LDL cholesterol (99 ± 27 mg/dL vs. 112 ± 29 mg/dL, p < 0.01) and serum HDL cholesterol (56 ± 13 mg/dL vs. 61 ± 16 mg/dL, p = 0.02) levels (Table 1).

### 3.3 The relations between thromboembolic risk, CKD, and CAD

**Fig. 3:**
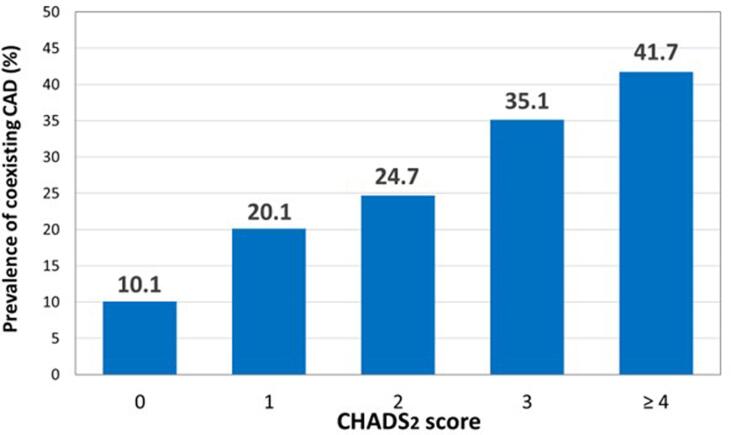
**Prevalence of CAD according to CHADS_2_ scores** A stepwise increase in CAD prevalence corresponding to the CHADS_2_ score is observed (10.1 % at 0 points, 20.1 % at 1 point, 24.7 % at 2 points, 35.1 % at 3 points, and 41.7 % at ≥ 4 points). CAD, coronary artery disease.

**Fig. 4:**
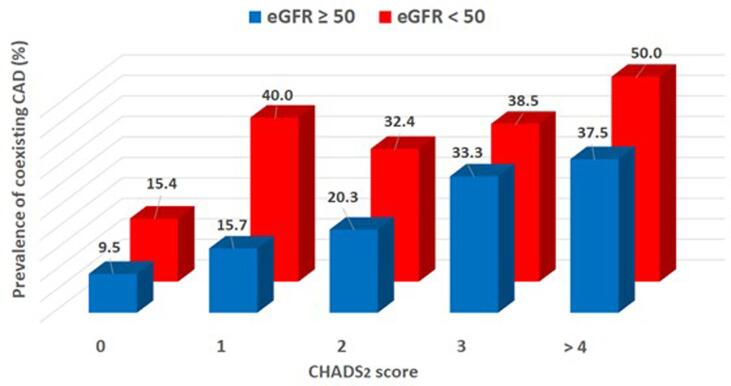
**Prevalence of coronary artery disease according to CHADS_2_ score and CKD.** An increased CAD prevalence corresponding to the CHADS_2_ score and CKD is observed. In patients with eGFR > 50 mL/min/1.73 m^2^, CHADS_2_ scores are 0 points in 11 of 116 patients (9.5 %), 1 point in 21 of 134 patients (15.7 %), 2 points in 12 of 59 patients (20.3 %), 3 points in 8 of 24 patients (33.3 %), and ≥ 4 points in 3 of 8 patients (37.5 %). In the patients with eGFR < 50 mL/min/1.73 m^2^, CHADS_2_ scores are 0 points in 2 of 13 patients (15.4 %), 1 point in 12 of 30 patients (40.0 %), 2 points in 11 of 34 patients (32.4 %), 3 points in 5 of 13 patients (38.5 %), and ≥ 4 points in 2 of 4 patients (50.0 %). CAD, coronary artery disease; eGFR, estimated glomerular filtration rate.

CAD prevalence showed a stepwise increase that corresponded with the CHADS_2_ score (Fig. 3). Scores were 0 points in 13 of 129 patients (10.1 %), 1 point in 33 of 164 patients (20.1 %), 2 points in 23 of 93 patients (24.7 %), 3 points in 13 of 37 patients (35.1 %), and ≥ 4 points in 5 of 12 patients (41.7 %). In the patients with eGFR ≥ 50 mL/min/1.73 m^2^, CHADS_2_ scores were 0 points in 11 of 116 patients (9.5 %), 1 point in 21 of 134 patients (15.7 %), 2 points in 12 of 59 patients (20.3 %), 3 points in 8 of 24 patients (33.3 %), and ≥ 4 points in 3 of 8 patients (37.5 %). In the patients with eGFR < 50 mL/min/1.73 m^2^, CHADS_2_ scores were 0 points in 2 of 13 patients (15.4 %), 1 point in 12 of 30 patients (40.0 %), 2 points in 11 of 34 patients (32.4 %), 3 points in 5 of 13 patients (38.5 %), and ≥ 4 points in 2 of 4 patients (50.0 %, Fig. 4).

## 4 Discussion

This study evaluated the prevalence of CAD in patients who underwent catheter ablation for AF and the clinical characteristics of these patients. The prevalence of CAD was 20 % in our cohort of patients with AF. Patients with CAD were older and had a greater prevalence of heart failure, hypertension, and diabetes mellitus, resulting in higher CHADS_2_ scores than those without CAD. Moreover, CAD prevalence showed a stepwise increase that corresponded with CHADS_2_ scores. Interestingly, impaired renal function was associated with an increased prevalence of CAD, suggesting that coincident CKD should be considered even in patients with low CHADS_2_ scores.

### 4.1 Assessment of coexisting CAD using coronary CT

The prevalence of coexisting CAD in patients with AF ranges from 14 % to 46.5 % [5], [6], [7], [8], [9], [10], which is consistent with the results of the present study. We defined CAD as stenosis ≥ 50 % on contrast-enhanced CT, as in previous reports [19], [20]. The usefulness of coronary CT was reported in the Coronary CT Angiography Evaluation for Clinical Outcomes: An International Multicenter (CONFIRM) Registry. In CONFIRM, obstructive CAD, defined as 50 % stenosis, was associated with higher rates of mortality in 23,854 patients without previously diagnosed CAD at 2.3 ± 1.1 years of follow-up compared with those without CAD. Furthermore, coronary CT revealed significant amounts of plaque, even at 50 % stenosis. In a study by Nakazawa et al., the CT density of the culprit plaque was lower, and a signet ring-like appearance was common in patients with a transient no-reflow phenomenon during subsequent PCI [23]. Currently, cardiac CT is often used to assess the anatomy of the left atrium and pulmonary veins before catheter ablation for AF and detect left atrial thrombus. It is considered a reliable alternative to transesophageal echocardiography and has been used more frequently during the coronavirus disease 2019 pandemic [24]. Our findings support the additional utility of cardiac CT for detecting coexisting CAD before catheter ablation for AF.

### 4.2 Management of coexisting CAD in patients with atrial fibrillation

Considering that new-onset AF increases the incidence of subsequent new-onset coronary ischemia events [15], and coexisting CAD increases the cardiovascular risk in patients with AF [5], [16], optimal medical therapy for existing CAD may be beneficial in patients with AF, irrespective of symptoms.

The International Study of Comparative Health Effectiveness with Medical and Invasive Approaches (ISCHEMIA) demonstrated that initial optimal medical therapy [25], including antiplatelet and statin therapy, was not associated with an increased risk of ischemic cardiovascular events or death from any cause over a median of 3.2 years in patients with stable coronary disease and moderate or severe ischemia compared with an initial interventional strategy. In the trial, strict guideline-based medical therapy goals were set, particularly targeting LDL cholesterol levels of < 70 mg/dL. Statins were recommended up to the maximum tolerated dose. If LDL cholesterol levels of < 70 mg/dL could not be achieved, ezetimibe and/or a proprotein convertase subtilisin/kexin type 9 inhibitor was recommended. This yielded a median LDL cholesterol level of 64 mg/dL at the final visit. A previous systematic review of 14 randomized controlled trials found that the use of statins for primary prevention was associated with reductions in all-cause mortality, major vascular events, and revascularizations in 34,272 participants without evidence of cardiovascular disease [26]. Furthermore, intensive statin treatment suppressed the progression of atherosclerosis, as assessed by intravascular ultrasound and coronary angiography [27]. This suggested that the effects of intensive statin therapy in reducing plaque volume may contribute to reduced major cardiovascular events in patients with CAD. Considering that patients with AF and high CHADS_2_ scores are expected to have coexisting CAD, intensive statin therapy may be beneficial in such patients without known coexisting CAD. In addition, current guidelines on lipid management in patients with CKD recommend statins or statin/ezetimibe for all individuals aged > 50  years with eGFRs < 60  ml/min/1.73  m^2^ to lower the risk of major cardiovascular events [28]. Therefore, lipid management should be considered among elderly patients with AF, particularly in those with impaired renal function.

In the ISCHEMIA trial, antiplatelet agents were mandatory for all patients. There is concern that the concomitant use of anticoagulants and antiplatelet agents may increase the risk of bleeding complications in patients with AF and coexisting CAD. The Atrial Fibrillation and Ischemic Events with Rivaroxaban in Patients with Stable Coronary Artery Disease (AFIRE) study demonstrated the non-inferiority of monotherapy with rivaroxaban (a non-vitamin K antagonist oral anticoagulant)—compared with combination therapy using rivaroxaban and a single antiplatelet agent—with respect to cardiovascular events and death from any cause and superiority with respect to major bleeding events [21]. Notably, the AFIRE study included both patients with prior PCI or CABG and medically treated patients who had not undergone interventional revascularization in whom CAD, defined as stenoses ≥ 50 %, was angiographically confirmed. However, to date, data regarding the feasibility and safety of monotherapy with other anticoagulants are scarce. Thus, further investigation is required to elucidate whether monotherapy with anticoagulants is appropriate for improving outcomes of patients with AF and coexisting CAD or whether combination therapy with antiplatelet agents is necessary.

### 4.3 Clinical implications

The present study demonstrated that 20 % of patients who underwent catheter ablation for AF had coexisting CAD; of these, 17.9 % were newly diagnosed with CAD. Interestingly, CAD prevalence increased with the CHADS_2_ score. As impaired renal function was associated with an increased prevalence of coincident CAD, the presence of CKD should be taken seriously in addition to the CHADS_2_ scores when stratifying the risk of coincident CAD. Given the high risk of subsequent coronary ischemic events in patients in whom AF is detected, special consideration should be given to potentially overlooked angina symptoms and the initiation of optimal medical therapy for coexisting CAD.

### 4.4 Study limitations

This study has limitations. First, in this retrospective single-center study, we enrolled patients with AF who underwent catheter ablation. This could imply a selection bias due to the exclusion of those who were not eligible for catheter ablation, such as the older population or patients with a long duration of persistent AF (≥5 years) [29]. Furthermore, we excluded 27 patients who did not consent to coronary angiography for calcified coronary arteries; this may have caused an underestimation of CAD prevalence. Second, no data regarding participants’ family history of CAD were available. Third, we investigated the prevalence of CAD using the CHADS_2_ score rather than the CHA_2_DS_2_-VASc score, which is more useful for stratifying the risk for cerebral infarction. However, considering that atrial fibrillation is now a common condition often encountered by general physicians other than cardiologists, we believe that stratification using the simplified CHADS_2_ score would more clearly and simply convey the importance of screening for coexisting CAD in patients at high risk for thromboembolic events to such general physicians. Finally, the long-term follow-up data of patients with coexisting CAD were unavailable. For these reasons, further studies are warranted to determine the long-term outcomes in more diverse groups of patients with AF and coexisting CAD in association with thromboembolic risk.

## 5 Conclusions

Coexisting CAD is present in one-fifth of the patients with AF. Notably, there is an association between CHADS_2_ scores and an increased CAD prevalence, which highlights the importance of screening coexisting CAD in patients at high risk for thromboembolic events, particularly in those with impaired renal function.

## Funding

This research did not receive any specific grant from funding agencies in the public, commercial, or not-for-profit sectors.

## CRediT authorship contribution statement

**Tsutomu Murakami:** Writing – original draft, Visualization, Validation, Supervision, Software, Resources, Project administration, Methodology, Investigation, Funding acquisition, Formal analysis, Data curation, Conceptualization. **Atsuhiko Yagishita:** Writing – review & editing, Writing – original draft, Visualization, Validation, Supervision, Resources, Project administration, Methodology, Investigation, Funding acquisition, Formal analysis, Data curation, Conceptualization. **Kengo Ayabe:** Data curation. **Susumu Sakama:** Data curation. **Kyong Hee Lee:** Data curation. **Mari Amino:** Writing – review & editing. **Koichiro Yoshioka:** Writing – review & editing. **Yuji Ikari:** Writing – review & editing.

## Declaration of competing interest

The authors declare that they have no known competing financial interests or personal relationships that could have appeared to influence the work reported in this paper.
